# Confirmatory Factor Analysis of the Malay-Language Transtheoretical Model of Physical Activity among Malaysian Primary School Children

**DOI:** 10.21315/mjms2019.26.2.11

**Published:** 2019-04-30

**Authors:** Hussein Rizal, Mawar Siti Hajar, Yee Cheng Kueh, Ayu Suzailiana Muhamad, Garry Kuan

**Affiliations:** 1 Exercise and Sports Science Programme, School of Health Sciences, Universiti Sains Malaysia, Kubang Kerian, Kelantan, Malaysia; 2 Unit of Biostatistics and Research Methodology, School of Medical Sciences, Universiti Sains Malaysia, Kubang Kerian, Kelantan, Malaysia

**Keywords:** Transtheoretical Model (TTM), physical activity, processes of change, decisional balance, self-efficacy, validity, confirmatory factor analysis (CFA), Malay, children

## Abstract

**Introduction:**

The transtheoretical model (TTM) is an integrative model of intentional change consisting of stages of change, processes of change, decisional balance and self-efficacy. This study aimed at validating the TTM questionnaires on physical activity for Malaysian children using confirmatory factor analysis.

**Methods:**

The participants were 381 Malay students (188 male; 193 female), aged 10–12 years old, with a mean age of 10.94 (SD = 0.81). The original version of the TTM was translated into the Malay language using forward and backward translation. Certain phrases were adapted based on the local culture and vocabulary suitable for primary school students.

**Results:**

The final measurement models and their fit indices were: processes of change (CFI = 0.939, TLI = 0.925, SRMR = 0.040, RMSEA = 0.030); decisional balance (CFI = 0.897, TLI = 0.864, SRMR = 0.045, RMSEA = 0.038); and self-efficacy (CFI = 0.934, TLI = 0.915, SRMR = 0.042, RMSEA = 0.032).

**Conclusion:**

Care must be taken when using the TTM with children, as it has been prevalently validated with adults. The final version of the TTM questionnaire for Malay primary school children had 24 items for process of changes, 13 items for self-efficacy and 10 items for decisional balance.

## Introduction

Physical activity is defined as any bodily movement that results in the expenditure of energy. It is well established that physical activity can reduce the risk of depression and non-communicable diseases, such as hypertension, obesity, cardiovascular diseases, diabetes and colon and breast cancer (1). According to a systematic review of recommendations for physical activity by Janssen and LeBlanc (2), children and youth 5–17 years of age should engage in an average of at least 60 min and up to several hours of moderate-intensity physical activity per day. Aerobic exercise should be the main component of daily physical activity, while muscle and bone strengthening should be included at least three days weekly.

Despite these numerous health benefits, Poh et al. (3) found that most Malaysians do not adequately participate in physical activity. Since 1991, the Malaysian Ministry of Health has conducted the Healthy Lifestyle Campaign with different themes, including promotion of exercise and physical activity (4). However, the campaign has shown little success as most Malaysian adults have remained generally sedentary, with only 14% reporting being adequately active (3). In addition, the Malaysian School-Based Nutrition Survey 2012 and Nutrition Survey of Malaysian Children classified that more than half of the children and adolescents in Malaysia as having low levels of physical activity and high levels of sedentary behaviour (5, 6). Malaysian children reportedly have a higher likelihood of being overweight as they age (7). The data indicate a need for a theory-based framework to improve physical activity participation and reduce sedentary behaviour in the Malaysian population.

The transtheoretical model (TTM) is an integrative model of intentional change of health behaviours developed based on numerous theories of the psychotherapy (8). The TTM has been successfully applied to a wide range of health behaviours, including physical activity, smoking-cessation and stop-drinking programmes. The four constructs of the TTM are stages of change, processes of change, decisional balance and self-efficacy (9). The TTM is aimed at assessing changes in individual across the stages of change and the related perceived pros, cons, self-efficacy and cognitive and behavioural adaption for physical activity. Stage-matched interventions use different strategies to induce physical activity behaviour change (10). These measures enable quantifying the stages of change and creating appropriate strategies to improve physical activity levels. For example, if a participant is in the lower stages of change (i.e. pre-contemplation or contemplation), then more cognitive approaches are used to improve the participant’s perceptions of and motivation for physical activity, such as promotional campaigns, counselling and introductory sport sessions. However, if the participant is in the higher stages of change (i.e. action or maintenance), then the participant is more inclined to do physical activity, so improvement strategies include adding or improving sports facilities and offering more physical activity programs.

The central construct of this model is the stages of change, which has a temporal dimension as individuals progress cyclically through six stages when attempting to change their behaviour (9). The six stages are pre-contemplation, contemplation, preparation, action, maintenance and termination. The further ahead individuals are in the stages of change, the more actively they are pursing or maintaining healthy behavioural change. The processes of change construct is divided into two higher-order factors: cognitive processes and behavioural processes. In theory, cognitive strategies, such as consciousness raising, dramatic relief, self-re-evaluation, environmental re-evaluation and self-liberation (11), are used in the earlier stages of change. When individuals are not actively striving to change their immediate health behaviour, cognitive adaptions can be used to alter their thoughts and motivate them to change. Later stages of change demand behavioural strategies, such as social liberation, counter-conditioning, stimulus control, reinforcement management and helping relationships (11). In the later stages of change, individuals actively pursue healthy behavioural change, so strategies to strengthen and maintain their healthy behaviour are important to avoid relapse. Velicer et al. (12) reported that cognitive processes are mostly involved in the early stages, and behavioural processes in the later stages of change. However, Dishman et al. (13) mentioned that many people appear to use both cognitive and behavioural processes while attempting to increase or maintain their physical activity. Nonetheless, cognitive strategies are used more frequently by those in the early stages, and behavioural strategies by those in the later stages (14).

In addition to the processes of change, the TTM construct includes decisional balance, or individuals’ weighing of the relative pros and cons of change (9). Originally developed by Janis and Mann (15), decisional balance has two factors: pros and cons. Generally, individuals perceive more barriers to changing their health behaviour during the early stages of change but gradually perceive more benefits as they progress through the stages (16). The final construct in the TTM is self-efficacy, which was originally developed by Bandura (17). Self-efficacy has three factors: internal feelings, situational demands and competing demands (18). Self-efficacy is characterised as situation-specific confidence that can be used to cope with high-risk situations without relapsing into unhealthy habits (19).

We found no published Malay version instrument measuring the TTM constructs (processes of change, decisional balance and self-efficacy) specifically for younger children or primary school student in Malaysia. However, it is important to understand the TTM constructs that influence younger children’s participation in physical activity in Malaysia. Furthermore, the TTM questionnaires are commonly used in studies on adult populations, and research on the validity of the questionnaires with younger children is limited. This study, therefore, was aimed to translating the TTM questionnaire into the Malay language and examining its reliability and validity among children and students studying at Malay primary schools.

## Methods

### Research Design

This cross-sectional study was conducted in a local primary school in the district of Kelantan, Malaysia. The purpose of this study was to validate the Malay language version of the TTM on physical activity, including questionnaires on processes of change, decisional balance and self-efficacy, specifically for Malay primary school children. The study was intended to give insights into the use of TTM on physical activity in the Malaysian setting.

### Participants and Sampling

A total of 381 students (188 males and 193 females) 10–12 years old (mean [SD] = 10.94 [0.81]) volunteered to participate. The students were all Malay and had engaged in at least one 30-minute session of physical and leisure activities in the past seven days. Confirmatory factor analysis (CFA) was performed as larger samples generally produce more stable solutions and are more likely to be replicable (20). A sample size of at least 200 was recommended for structural equation modelling in studies involving CFA (21). A total of 420 students in years 4–6 of primary school volunteered for the study. However, 19 did not completely answer all the questionnaires, and 20 did not submit signed, parental informed consent forms, so they were excluded. The sample size (*N* = 381) was adequate for the confirmatory study.

### Questionnaire Translation

The original English language version of the TTM was translated into the Malay language in the following steps. First, one author forward translated the English version into Malay, retaining the meanings and considering the children’s vocabulary level. Second, a local Malay lecturer bilingual in Malay and English back-translated the Malay version to English. Third, two experienced lecturers in sports sciences, sports psychology and physical activity reviewed the English translation from Malay and the Malay translation from English and compared each item to its corresponding item in the original English version. They also assessed whether the contents were compatible with Malaysian culture and noted any deviations in meaning. Fourth, a pre-test of the final version of the TTM was carried out with a class of thirty 12-year-old students at Sekolah Kebangsaan Seri Kota, Kota Bharu, Kelantan. They were asked to answer the questions and comment on the wording of the questionnaires. The pre-test results indicated a need for several changes in the wording and presentation to make it easier for the students to answer.

### Measures

#### Stages of change

The stages of physical activity adoption was developed by Marcus et al. (21). For six statements in the questionnaire, the respondents chose the one best representing their current stage of physical activity. Progressing through the stages from pre-contemplation to termination generally has increased the amount of regular physical activity in which individuals engage. Test re-test reliability was reported to be 0.78 (21).

#### Processes of change

The processes of change construct was developed by Nigg et al. (22). In this self-administered 30-item questionnaire with a 5-point Likert scale, the respondents were asked to answer items based on their preferences ranging from ‘never’ to ‘repeatedly’. This construct measured 10 factors: consciousness raising, dramatic relief, self-re-evaluation, environmental re-evaluation, social liberation, counter-conditioning, helping relationships, reinforcement management, stimulus control and self-liberation. The internal consistency reliability was reported to be 0.6 and 0.9 for the two higher factors, cognitive and behavioural processes, respectively (22).

#### Decisional balance

The decisional balance construct was developed by Plotnikoff et al. (23). In this self-administered 10-item questionnaire with a 5-point Likert scale, the respondents were asked to answer the items based on their preferences ranging from ‘not at all’ to ‘extremely’. The two factors in this scale were pros and cons, representing the positives and negatives of physical activity. The internal consistency reliability was reported to be 0.82 and 0.72 for pros and cons, respectively (23).

#### Self-efficacy

The self-efficacy in exercise construct was developed by Bandura (17). The revised version by Kim (24) was used in the present study. In this self-administered 18-item questionnaire with a 5-point Likert scale, the students were asked to answer the items based on their preferences ranging from ‘cannot do’ to ‘certainly can do’. Self-efficacy had three factors: internal feelings, situational demands and competing demands. Test re-test reliability was reported to be 0.86 (24).

#### Leisure-time exercise questionnaire

The Leisure-time Exercise Questionnaire (LTEQ) was developed by Godin and Shepard (25), was used to assess habitual, weekly physical-activity behaviour. In this exercise, the respondents completed a self-explanatory, brief, 4-item query on their usual leisure-time exercise habits. Data were presented as physical activity frequency (always, sometimes and rarely). In addition, the metabolic equivalent of tasks (METs), which was the ratio of the work metabolic rate to the resting metabolic rate, was calculated by multiplying strenuous exercise by nine, moderate exercise by five and mild exercise by three. These scores were all totalled to calculate the total METs.

#### Procedures

Data collection was conducted in Sekolah Kebangsaan Seri Wakaf Bharu, Kelantan. Convenience sampling was used to recruit students from classes not taking major school or national exams. The study was approved by the Research Ethics Committee of Universiti Sains Malaysia (USM/JEPeM/18020104) and conducted in accordance with the Declaration of Helsinki. Approval was also obtained from the Malaysian Ministry of Education, Kelantan State Education Department and schools. The study followed a two-step validity process; content validity was checked by the translation process, and construct validity was checked via CFA.

Students were given consent and assent forms for their parents to read, comprehend and sign to allow their children to participate in this study. The students were informed that their participation was voluntary and that they could withdraw from the study at any time without any loss of benefits to which they were entitled. The questionnaires were administered to the students during school hours, and was conducted in the school’s main hall. Before the students completed the questionnaires, each section was clearly explained to them. The students took approximately an hour to complete the questionnaires. Once they finished, the students were given an honorarium in the form of a certificate as a sign of gratitude for participating in the study. The study results were also shared with the school.

#### Data analysis

Statistical analysis was carried out using Mplus version 8 and SPSS version 22. Data were screened for missing values before the analysis. Nineteen students had more than five missing values, so they were excluded from the study. Another 20 students did not have signed informed consent forms, leaving a total of 381 questionnaires for analysis. The numerical data was reported as means and standard deviations, and the categorical data as frequencies and percentages. Descriptive analysis and Cronbach’s alpha were conducted using SPSS. A CFA function is not available in the SPSS package, so CFA and composite reliability was conducted using Mplus. The following fit indices were used to assess the CFA model fit: comparative fit indices (CFI) and Tucker and Lewis index (TLI) with a desired value of more than 0.92; and root mean square error of approximation (RMSEA) and standardised root mean square (SRMR) with a desired value of less than 0.08 (26). Furthermore, Hair et al. (26) suggested that a good standardised loading factor of each measurement’s latent variable quantified from manifest variables should be more than 0.50 and ideally 0.70 or higher. Regarding reliability, Nunnally (28) recommended a minimum of 0.70 as the cut-off point for acceptable Cronbach’s alpha. However, due to Cronbach’s alpha limitations, it was technically more appropriate to apply a different measure of internal consistency reliability considering the different outer loadings of the indicator variables: composite reliability, with a desired value of more than 0.6 (27). Both reliability measures, therefore, were used in the study.

## Results

Table 1 presents the demographic information and frequency of the students’ stages of change. Of the 381 participants, 188 (49.3%) were male, and 193 (50.7%) female. Regarding physical activity frequency, 137 (36.0%) of the students were physically active, 228 (59.8%) were sometimes active, and 16 (4.2%) were not active. The mean and standard deviation of the total METs calculated was 70.8 (24.05). Of the 381 students, 10 (2.6%) were in the pre-contemplation stage, 30 (7.9%) in the contemplation stage, 185 (48.6%) in the preparation stage, 32 (8.4%) in the action stage, 109 (28.6%) in the maintenance stage, and 15 (3.9%) in the termination stage.

The measurement model in Table 2 shows several models adjusted to gain the best fit. The fit indices for the initial model for processes of change were not within the recommended values. To obtain better fit, the factors of dramatic relief (items 4, 5 and 6) and environmental re-evaluation (items 7, 8 and 9) were removed after obtaining adequate theoretical support. The final model for processes of change fit well based on several indices: CFI = 0.939, TLI = 0.925, SRMR = 0.040, RMSEA = 0.030 (90%CI = 0.02–0.04), RMSEA *P*-value = 1.00. The initial model for decisional balance also fit the data well: SRMR = 0.045, RMSEA = 0.038 (90%CI = 0.014–0.057), RMSEA *P*-value = 0.844. Although CFI (0.897) and TLI (0.864) indicated that the model did not fit the data well, the model for decisional balance was not further modified as the items in decisional balance were found to be suitable and important for the study population. The initial model for self-efficacy showed very poor fit (CFI = 0.669, TLI = 0.617) and required modification. After adequate theoretical support was carried out, the error correlations of several items, including items 4 with 8, 12 with 13, and 8 with 15, were iteratively added to the model. Low-loading items 4, 5, 9, 12 and 16 were removed to acquire better fit. The final model for self-efficacy had good fit based on several fit indices: CFI = 0.934, TLI = 0.915, SRMR = 0.042, RMSEA = 0.032 (90%CI = 0.011–0.047), RMSEA *P*-value = 0.977.

**Figure 1:**
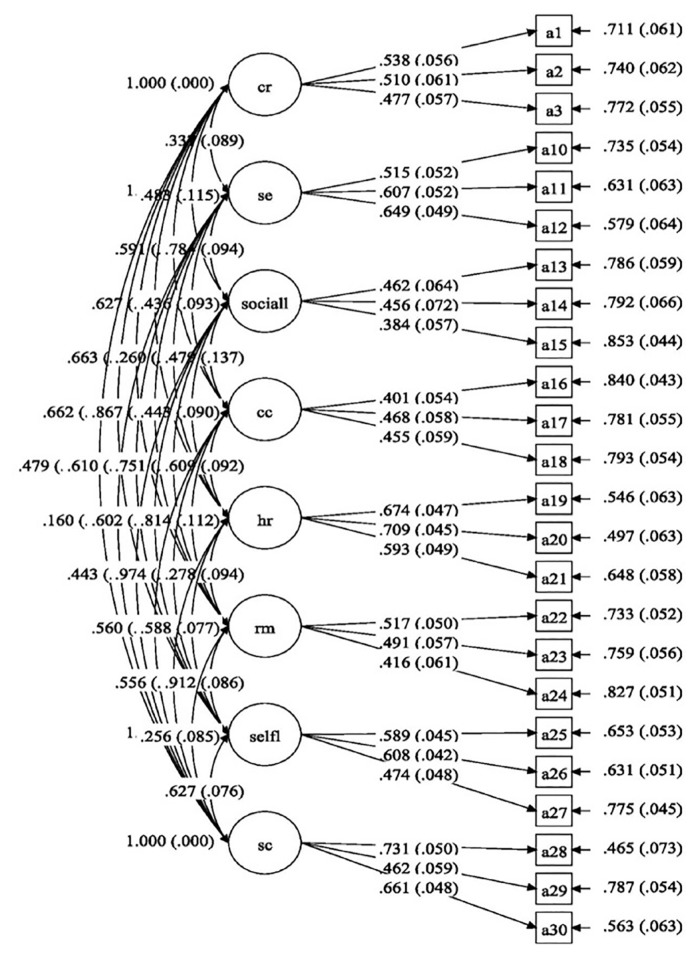
Standardised item loading for the measurement model of processes of change

Figure 1 shows the standardised item loading for the measurement model of process of change. The diagram represented is the final result after removing problematic items based on the CFA results and gaining adequate theoretical support. After removal of items 4–9, the item loadings were acceptable, except for item 15, which had a factor loading of 0.384. The removed items involved the factors of dramatic relief and environmental re-evaluation.

**Figure 2:**
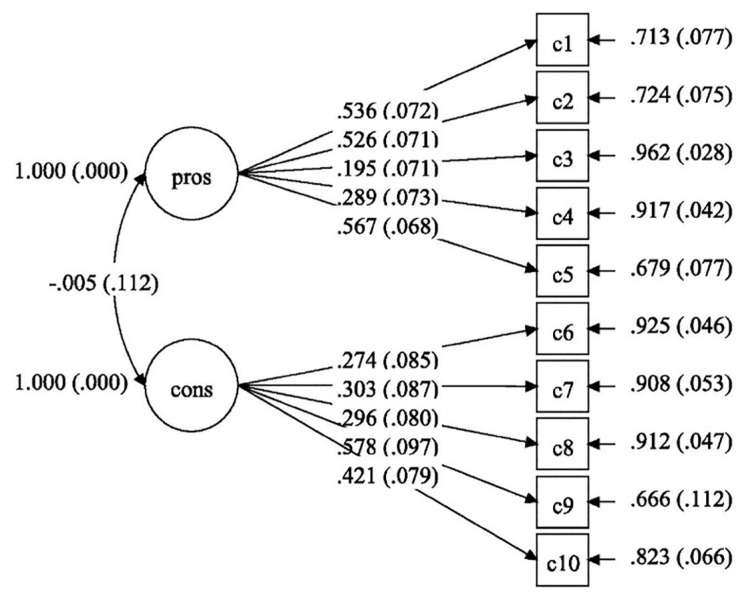
Standardised item loading for the measurement model of decisional balance

Figure 2 presents the standardised item loading for the measurement model of decisional balance. The model had good factor loading for items 1, 2, 5, 9 and 10 (0.421–0.578). The values for items 3, 4, 6, 7 and 8 (0.195–0.303) were below the cut-off point for acceptable factor loading. The items were not removed, however, to maintain representation of the model for pros and cons. Removing these items would have influenced the theoretical meaning of the construct.

**Figure 3:**
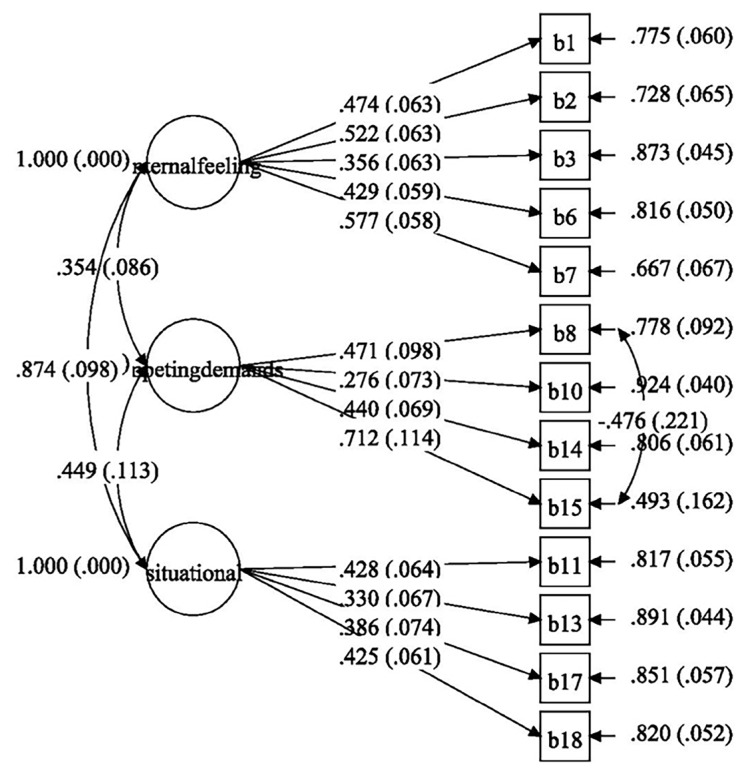
Standardised item loading for the measurement model of self-efficacy

Figure 3 gives the standardised item loading for the measurement model of self-efficacy. The diagram represented is the final result after correlating residuals and removing items. After removal of items 4, 5, 9, 12 and 16, the factor loadings were acceptable (0.425–0.712). Items 3, 10, 13 and 17 had low factor loadings (0.276–0.386) but were not removed as it would affect the theoretical construct of the TTM, specifically the self-efficacy construct. After re-examining the meaning of these items, we deemed them suitable for Malay primary school children.

Table 3 displays the changes in standardised item loadings after item removal in the process of change model. Items 4, 5, 6, 8 and 9 had low factor loadings (0.186–0.324). Hair et al. (28) recommended a minimum acceptable factor loading of 0.4. The factors of dramatic relief (item 4 = 0.186, item 5 = 0.324, item 6 = 0.228) and environmental re-evaluation (item 7 = 0.507, item 8 = 0.292, item 9 = 0.203) had low factor loadings and were removed to gain better fit model. Although item 7 had acceptable loading (0.507), it had to be removed as a single factor could not be representative. This process resulted in a model of 8 factors with 24 items. Item 15 subsequently had a low factor loading but was kept in the model to avoid misrepresentation of the factor. Any factors with fewer than 3 items were not stable based on the CFA results.

Table 4 shows the changes in standardised item loadings after performing correlation and item removal for the self-efficacy model. Correlation of residuals was conducted for items 4 with 8, 12 with 13, and 8 with 15. Next, items 4, 5, 16, 12 and 9 were removed based on the lowest factor loading. The final result provided less desirable loading as four items had values less than the cut-off point for acceptable factor loading. However, removing any more items would reduce the factor loading for item 14 from an acceptable level (0.440) to an unacceptable level (0.394). Therefore, only items 4, 5, 16, 12 and 9 were removed. Finally, the composite reliability factors in processes of change, decisional balance and self-efficacy ranged from 0.347 to 0.710, while Cronbach’s alpha values ranged from 0.397 to 0.694. Table 5 reports the reliability scores.

## Discussion

The study aim was to determine the validity and reliability of the TTM Malay version questionnaires for Malaysian children. This study combined three distinct questionnaires on processes of change, decisional balance and self-efficacy as it was important to measure these changes across the stages of change. However, due to the large scope of measuring the changes in the three constructs across the stages of change (stages of change algorithm) and calculating the construct validity and internal reliability of each construct, the authors believed that it was sufficient to analyse the latter via CFA in the Malaysian setting. Moreover, to the authors’ knowledge, fewer validation studies have used CFA with younger populations than have measured these constructs across the stages of change (29). Nonetheless, future studies are needed to explore the stages of change algorithm when used with physical activity in the Malaysian setting.

The processes of change measured overt (experiential) and covert (behavioural) changes to individuals’ health behaviour. For adults, most studies have replicated a good fit for the model (29–33). However, Dishman et al. (13) tested the model for processes of change among college students and found that it showed poor fit and required extensive modifications, such as removal of the self-liberation factor. In the present study, the factors of dramatic relief and environmental re-evaluation had low factor loadings and were removed (Table 3). Dramatic relief might not have been relatable to the children who, at their age, had not experienced any events forcing them to make healthy behavioural changes. Item 4 (“I get upset when I see people who would benefit from exercise but choose not to exercise”), item 5 (“I am afraid of the consequences for my health if I do not exercise”) and item 6 (“I get upset when I realise that people I love would have better health if they exercised”) reflected the thoughts of more mature individuals with past experience of the consequences of physical inactivity. A systematic review by Allender et al. (34) concluded that children’s motivations for physical activity were experimentation, unusual activities, parental support and safe environments. Children found participation to be more enjoyable when they were not forced to compete and win but were encouraged to experiment with different activities (34). As children grew older, their motivations to be more physically active evolved to suit their new needs: for adolescents, body shape maintenance, weight management, creation of new social networks, family support and peer support; and for adults, a sense of achievement, skills development, medical sanction, health benefits and enjoyment (35–41). In short, children were more open to participating in physical activity without a need or reason for change. Accordingly, the results indicated low factor loadings for the items for dramatic relief.

The environmental re-evaluation factor was also removed due to low factor loadings. Only two of its three items showed low factor loading, but it could not be represented by only one item, so it was removed. Item 7 (“I realise that if I don’t exercise regularly, I may get ill and be a burden to others”) was a straightforward question that students could easily understand and showed acceptable factor loading (Table 4). Item 8 (“I think that exercising regularly will prevent me from being a burden to the healthcare system”) and item 9 (“I think that regular exercise plays a role in reducing health care costs”) arguably were not relatable questions for the children and consequently had very low factor loadings. These questions might have been better suited for older participants with more knowledge of the importance of health and Malaysia’s dichotomous, public–private health care system (42). To the authors’ knowledge, no studies have assessed the structural content of processes of change among children. Studies evaluating construct and criterion validity targeted at children have been prioritised (28) over CFA studies. That is, the stages of change algorithm has been compared to previously recommended stages of change scales constituting the gold standard in TTM studies (42).

Decisional balance, another construct in the TTM, represented the pros and cons of adopting more physical activity for individuals’ lifestyles. In similar studies on adults, a two-factor, 10- item questionnaire showed good validity and reliability (23, 43). In the present study, the decisional model showed adequate fit (44) but fell slightly below the cut-off point for current trends in fit-based indices, set at 0.90 for CFI and TLI (24). In a study by Uechi et al. (45) on decisional balance among Japanese elementary school children, a two-factor construct consisting of nine items showed high validity and reliability. In another study by Carlson et al. (46), CFA results demonstrated good fit for both the pros and cons, based on four items each. However, the construct validity for the pros were not supported. The present study’s validation results for decisional balance scale differed from previous studies, perhaps as the age group of 10–year-old children was younger than those studied previously (e.g. 23, 43, 46). To the authors’ knowledge, few CFA studies on decisional balance scale targeted at children have been conducted.

The study results showed low factor loadings for items 3, 4, 6, 7 and 8 (0.195, 0.289, 0.274, 0.303 and 0.296, respectively). Item 3 (“Physical activity would help me sleep better”) and item 4 (“Physical activity would help me have a more positive outlook”) represented the pro factor. Item 6 (“I am too tired to be physically active because of my other daily responsibilities”), item 7 (“Physical activity would take too much of my time”) and item 8 (“I would have less time for my family and friends if I participated in physical activity”) represented the cons factor. These factors may have been relevant to adult and college-age populations, but the children might not have had sufficient responsibility to make decisions based on the pros and cons of exercise. A revised model of decisional balance provided sufficient evidence among genders and ethnicities but not age groups (46). For example, items 6, 7 and 8 depicted physical activity as taking away time from other responsibilities and family. The children, though, spent most of their time with their families and were not as burdened by responsibilities and expectations as adults or even adolescents. In addition, items 3 and 4 representing the pros of physical activity may have been less understandable to the children. They might not have understood the relationship of physical activity with better sleep and positive outlooks unless they had some knowledge of sports science or had experienced the benefits of physical activity for their sleep and outlook. However, removing these many items would have misrepresented the two-factor construct. Previous studies have shown good construct validity even among children (45) but could vary across cultures (47). Further studies focusing on decisional balance, rather than the entire TTM construct, among children in the Malaysian population are needed.

The exercise self-efficacy scale developed by Bandura (17) was revised for the Korean version applied by Kim (43) to an adult population. In this study, the self-efficacy scale was translated into Malay and phrased to suit the younger population. Nine of the 18 items showed low factor loading (0.233–0.386). However, only five were removed as doing would reduce the factor loadings for item 14, which fell below the cut-off point after removing the item with the lowest factor loading, decreasing from 0.440 to 0.394. In addition, the original model had less than the required fit indices for good fit and required extensive modifications, particularly correlation and removal, to obtain a final good-fit model: CFI = 0.934, TLI = 0.915, RMSEA (90%) = 0.032 (0.011–0.047), *P*-value = 0.977. Previous studies showed a good-fit model for a three-factor, 18-item scale (24, 43) among adult populations. To the authors’ knowledge, few CFA studies have been conducted among younger populations. Instead of multiple questionnaires, future studies should administer a single questionnaire during sessions, especially to maintain children’s interest and avoid insincere answers.

Regarding reliability, Hair et al. (26) suggested that models had different fit indices depending on the sample size and the number of observed variables. In addition, composite reliability was prioritised over Cronbach’s alpha as the former considered the different outer loadings of the indicator variables (28), which Cronbach’s alpha did not detect. Nonetheless, both types of reliability were reported in this study as previous validation studies prioritised reporting Cronbach’s alpha over composite reliability. In this study, the reliability of the entire TTM construct was 0.347–0.710 for composite reliability, while Cronbach’s alpha was 0.397–0.629. Based on the cut-off point for both reliability measures proposed by Hair et al. (26), the study results showed poor reliability, except for helping relationships and stimulus control in the processes of change construct. However, previous TTM studies among adult populations showed good reliability (25, 43). In the present study on primary school students, the reliability values were low for most of the constructs, perhaps due to the low number of items for each factor. For example, each factor in the processes of change only had three items, leaving room for more unreliability. In addition, due to the multiple questionnaires (total = 58 items) administered during the session, the students might have felt less inclined to answer sincerely, leading to inconsistent answers and reducing the reliability of the tests. In future studies, the Malay version of the TTM questionnaires should be revisited, and new items can be added to the constructs with fewer items (e.g. three items per factor in processes of change). Some items may need to be revised to maintain consistent meanings with other items in the same factor.

A main limitation of this study was the sheer number of items in the questionnaires. The TTM had 58 items, excluding Godin’s leisure-time exercise scale and the stages of change. Most of the items were for processes of change (30 items), followed by self-efficacy (18 items) and decisional balance (10 items), based on previous validation studies conducted with Asian adults and adolescent populations (10–11, 48). The 10–12-year-old children found it difficult to concentrate and answer the questions on their own and needed constant supervision. Conducting the session in the school hall with all 381 students created a noisy environment, which may have increased candidate-related errors. Furthermore, the instructions and wordings, especially on the processes of change questionnaire, were lengthy, which might have discouraged the children’s interest in answering honestly. In addition, some children could not understand terms used frequently in the questionnaire (e.g. commitment, awkward and goals), which warrant further investigation in future studies. Some problematic items were deemed to be unsuitable for children, and some should be rephrased to match children’s vocabulary level. In addition, the primary school teachers only permitted collecting data from students in year 4–6 not taking major examinations during the data collection period. Consequently, participants in the 10–12-yearold age group became the focus of the study. Future validation studies should include children older than age 12 and secondary school students. Doing so could allow generalising the validated Malay version TTM questionnaires to wider age groups of children in Malaysia.

## Conclusion

The validity and reliability of the Malay version of the TTM questionnaires (process of change, decisional balance and self-efficacy) for physical activity among Malay primary school children were tested using CFA. This study provided insights into the use of the TTM for physical activity in the Malaysian setting. Extensive modifications, particularly correlating the items’ residuals and removing problematic items, were needed to improve the model for younger children. The final version of the TTM questionnaires for Malay primary school children had 24 items for process of changes, 13 items for self-efficacy and 10 items for decisional balance. A total of 47 (81%) of 58 TTM items were retained. The shortened version of the Malay version of TTM questionnaires was considered to be valid, and the remaining items were found to be suitable for Malay primary school children.

## Figures and Tables

**Table 1:** Demographics and stages of change of school children (*N* = 381)

Demographic	Mean (SD)	Frequency (%)

Age	10.94 (0.81)	
Gender:		
Male		188 (49.3)
Female		193 (50.7)
Physical activity frequency:		
Always		137 (36.0)
Sometimes		228 (59.8)
Rarely		16 (4.2)
Weekly physical activity (METs)	70.8 (24.05)	
Stages of change:		
Pre-contemplation		10 (2.6)
Contemplation		30 (7.9)
Preparation		185 (48.6)
Action		32 (8.4)
Maintenance		109 (28.6)
Termination		15 (3.9)

**Table 2:** Model fit indices for processes of change, decisional balance and self-efficacy

Construct	Model	CFI	TLI	SRMR	RMSEA (90%CI)	RMSEA *P*-value
Processes of change	Model-1 (Original)	0.905	0.885	0.050	0.034 (0.027 – 0.040)	1.000
	Model-2 (Removed items 4–9)	0.939	0.925	0.040	0.030 (0.022 – 0.040)	1.000
Decisional balance	Model-1 (Original)	0.897	0.864	0.045	0.038 (0.014 – 0.057)	0.844
Self-efficacy	Model-1 (Original)	0.669	0.617	0.064	0.062 (0.054 – 0.071)	0.010
	Model-2 (CR^1^ 4 with 8)	0.728	0.683	0.061	0.057 (0.048 – 0.065)	0.109
	Model-3 (CR^1^ 4 with 8 & 12 with 13)	0.751	0.707	0.060	0.054 (0.045 – 0.063)	0.205
	Model-4 (CR^1^ 4 with 8, 12 with 13 & 8 with 15)	0.776	0.734	0.059	0.052 (0.043 – 0.061)	0.368
	Model-5 (Removed items 4, 5, 9, 12 & 16)	0.934	0.915	0.042	0.032 (0.011 – 0.047)	0.977

**Table 3:** Standardised item loadings before and after removal of items 4 to 9 for model process of change

Correlation & removal	Standardised item loading																													

	Consciousness Raising			Dramatic Relief			Environmental Re-evaluation			Self-Re-evaluation			Social Liberation			Counter-conditioning			Helping Relationship			Reinforcement Management			Self-liberation			Stimulus Control		
									
	1	2	3	4	5	6	7	8	9	10	11	12	13	14	15	16	17	18	19	20	21	22	23	24	25	26	27	28	29	30
Original	0.492	0.542	0.496	0.186^1^	0.324^1^	0.228^1^	0.507	0.292^1^	0.203^1^	0.510	0.584	0.669	0.445	0.452	0.402	0.402	0.471	0.449	0.682	0.708	0.584	0.519	0.498	0.404	0.587	0.607	0.478	0.732	0.461	0.661
Remove items 4–9	0.538	0.510	0.477	-	-	-	-	-	-	0.515	0.607	0.649	0.462	0.456	0.384^1^	0.401	0.468	0.455	0.674	0.709	0.593	0.517	0.491	0.416	0.589	0.608	0.474	0.731	0.462	0.661

1 Item with low factor loading

**Table 4:** Standardised item loading before and after correlation/removal of items for model self-efficacy

Correlation	Standardised item loading																	

	Internal Feeling						Competing Demands						Situational					
	
	1	2	3	5	6	7	9	4	8	10	14	15	11	12	13	16	17	18
Original	0.447	0.533	0.354^1^	0.233^1^	0.434	0.535	0.306^1^	0.386^1^	0.437	0.352^1^	0.435	0.442	0.436	0.374^1^	0.432	0.279^1^	0.400	0.359^1^
4 with 8;	0.451	0.539	0.351^1^	0.223^1^	0.439	0.532	0.300^1^	0.231^1^	0.267^1^	0.370^1^	0.433	0.555	0.450	0.354^1^	0.430	0.291^1^	0.385^1^	0.369^1^
4 with 8; 12 with 13;	0.457	0.538	0.352^1^	0.217^1^	0.439	0.532	0.298^1^	0.225^1^	0.253^1^	0.362^1^	0.429	0.573	0.451	0.287^1^	0.372^1^	0.275^1^	0.381^1^	0.398^1^
4 with 8; 12 with 13; 8 with 15;	0.462	0.534	0.354^1^	0.216^1^	0.433	0.532	0.299^1^	0.209^1^	0.432	0.314^1^	0.459	0.648	0.450	0.288^1^	0.364^1^	0.252^1^	0.389^1^	0.405
**Remove**																		
B4	0.463	0.533	0.355^1^	0.215^1^	0.432	0.531	0.301^1^	–	0.462	0.296^1^	0.447	0.685	0.451	0.289^1^	0.361^1^	0.250^1^	0.390^1^	0.407
B4 B5	0.470	0.537	0.350^1^	–	0.429	0.542	0.284^1^	–	0.460	0.291^1^	0.442	0.698	0.451	0.283^1^	0.356^1^	0.245^1^	0.389^1^	0.414
B4 B5 B16	0.476	0.532	0.346^1^	–	0.424	0.545	0.286^1^	–	0.487	0.274^1^	0.436	0.714	0.419	0.267^1^	0.327^1^	–	0.390^1^	0.410
B4 B5 B16 B12	0.474	0.523	0.353^1^	–	0.428	0.550	0.280^1^	–	0.479	0.273^1^	0.437	0.717	0.427	–	0.331^1^	–	0.388^1^	0.424
B4 B5 B16 B12 B9	0.474	0.522	0.356^1^	–	0.429	0.577	–	–	0.471	0.276^1^	0.440	0.712	0.428	–	0.330^1^	–	0.386^1^	0.425

1 Item with low factor loading

**Table 5:** Reliability scores for each factor of the TTM construct

Construct	Factor	Composite reliability	Cronbach’s alpha
Processes of change	Consciousness raising	0.486	0.493
	Dramatic relief	-	-
	Environmental re-evaluation	-	-
	Self-re-evaluation	0.561	0.617
	Social liberation	0.347	0.397
	Counter-conditioning	0.461	0.406
	Helping relationship	0.710	0.694
	Reinforcement management	0.553	0.480
	Self-liberation	0.588	0.579
	Stimulus control	0.675	0.629
Decisional balance	Pros	0.520	0.504
	Cons	0.459	0.449
Self-efficacy	Internal feeling	0.590	0.580
	Competing demands	0.503	0.443
	Situational	0.498	0.431
